# Health and Health Care Access of Autistic Transgender and Nonbinary People in Canada: A Cross-Sectional Study

**DOI:** 10.1089/aut.2023.0024

**Published:** 2025-02-05

**Authors:** Noah Adams, Kai Jacobsen, Lux Li, Matt Francino, Leo Rutherford, ChrŸs Tei, Ayden Scheim, Greta Bauer

**Affiliations:** ^1^ Ontario Institute for Studies in Education, University of Toronto, Toronto, Canada.; ^2^ Carleton University, Ottawa, Canada.; ^3^ Western University, London, Canada.; ^4^ Diversity Essentials, Ottawa, Canada.; ^5^ University of Victoria, Victoria, Canada.; ^6^ Our_Community Health Initiative, Vancouver, Canada.; ^7^ Drexel University, Philadelphia, Pennsylvania, USA.

**Keywords:** transgender, autism, COVID 19, Trans PULSE, Canada, community-based research

## Abstract

**Background:**

The existence and health care needs of individuals who are both autistic and transgender and nonbinary (TNB) are increasingly discussed publicly. While research demonstrating a greater prevalence of autism among TNB individuals continues to grow, little captures their experiences with primary, mental health, and gender-affirming care (GAC), particularly between self-identified and diagnosed autistic TNB individuals. This article explores this nexus.

**Methods:**

We conducted a subgroup analysis of the Trans PULSE Canada 2019 national survey (*n* = 2873). We included both individuals who self-reported a diagnosis of autism (*n* = 230) and those who self-identify as autistic without one (*n* = 176). We compared participant demographics, health status, and health care experiences by autistic status (diagnosed, self-identified only, or allistic [non-autistic]) using weighted chi-square tests and logistic regression analyses.

**Results:**

Of Trans PULSE participants, 14.3% were autistic (8.1% diagnosed, 6.2% self-identified). Compared with their allistic peers, autistic participants were younger, had lower levels of education, employment, and income, and were more likely to identify as asexual. They also reported worse overall general health, a higher rate of unmet health care needs, and significant mental health disparities. While few diagnosed (3.7%) or self-identified (1.1%) autistic participants reported being directly denied GAC due to autism, 25.5% of diagnosed and 36.1% of self-identified individuals preemptively avoided sharing information about it during GAC readiness assessments in the past year.

**Conclusions:**

Our findings highlight the need for changes to treatment of autistic TNB people in primary and mental health care. Future research should explore both individual responses and systematic changes to these challenges.

## Abstract (plain language summary)

Community brief

Why is this an important issue?

Our research team is primarily made up of neurodivergent transgender and nonbinary (TNB) individuals. We wanted to better understand the experiences and challenges faced by people who are both TNB and autistic (autistic TNB). Research on this experience is relatively rare, and little is created by those with lived experience of this intersection. Without this information, gender-affirming care (GAC) providers often treat autistic TNB people overcautiously, for instance, by requiring longer waiting periods to access care.

What was the purpose of this study?

Our study compares health and health care experiences of autistic (both diagnosed and self-identified) and allistic (non-autistic) TNB people in Canada.

What did the researchers do?

We report on a subanalysis of the 2019 Trans PULSE Canada survey on the health and well-being of 2873 TNB people in Canada. Our study explored the health and health care experiences of the 406 participants who indicated that they were diagnosed with autism or self-identified as autistic when asked. We conducted statistical analyses to compare the responses of autistic TNB individuals with allistic participants in the wider Trans PULSE Canada study.

What were the results of the study?

Autistic individuals in our study were slightly younger and had lower reported levels of education, employment, and income than allistic participants. They were also much more likely to identify as asexual. Overall, autistic participants reported worse general and mental health and experienced more unmet health care needs than those who were allistic. Very few diagnosed (3.7%) or self-identified (1.1%) autistic participants told us that they were denied GAC due to autism. However, 25.5% of diagnosed respondents and 36.1% of self-identified respondents reported avoiding sharing information about their autism during GAC readiness assessments. Furthermore, those with an autism diagnosis were more likely than either self-identified or allistic respondents to have difficulty affording GAC (45.6% vs. 33.0% vs. 32.5%). This group was also more likely to have been denied GAC due to a disability other than autism (5.5% vs. 1.1% vs. 1.2%).

What do these findings add to what was already known?

Our findings help to fill gaps in what is known about autistic TNB people's experiences of health care. They also help to clarify how autistic people are treated when seeking GAC. Our study found, for instance, that autistic TNB people are misgendered more often by mental health care providers than allistic TNB individuals. This information could be used to provide training on making TNB mental health care practice more accessible and welcoming to autistic people. Similarly, future research could further explore why such misgendering seems to happen more often with autistic TNB individuals.

What are potential weaknesses in the study?

Our research did not use a random sample and so our results are not representative of all autistic TNB people in Canada. We also did not include any cisgender autistic people in our study. As a result, we cannot draw any conclusions about the comparative experiences of TNB and cisgender autistic people.

How will these findings help autistic adults now or in the future?

Autistic TNB participants in our study experienced multiple barriers to accessing health care, had worse overall health than allistic TNB people, and showed a tendency to hide their autism when attempting to access GAC. We hope that these findings can help to reduce health care barriers and disparities for autistic TNB individuals and communities. Autistic TNB people need and deserve access to GAC for the same reasons and on the same terms as allistic individuals.

## Introduction

Academic and public discourse on autistic and transgender and nonbinary (TNB) experiences has increased dramatically in recent years, with several academic publications citing a greater proportion of individuals at this intersection.^1–3^ A recent meta-analysis, for instance, estimated that 11% of people with gender dysphoria have a diagnosis of autism spectrum disorder.^4^ Individuals at this intersection (autistic TNB) are known to experience substantial barriers to health care, such as low socioeconomic status, underemployment, and stigmatization.^5–10^ These issues compound difficulties already experienced by autistic individuals (such as provider ignorance of sensory, communication, and executive function needs)^10–12^ and those specific to TNB individuals (such as transphobia and access to gender-affirming care [GAC]).^13–15^ Existing data on how these factors intersect within the Canadian TNB population are, however, limited.

Furthermore, little is known about the ways that the multiple health care barriers faced by autistic TNB individuals compare between self-identified and formally diagnosed autistic individuals.^16^ Existing publications appear to compare health experiences and TNB status separately, or not at all,^17^ or else explore the phenomenon of self-identification itself.^18^ Our study helps to close this gap by exploring Canadian autistic TNB people's experience of primary, mental health, and GAC using the Trans PULSE^19^ data set. We then compare these findings with the experiences of allistic (non-autistic) TNB individuals, as well as between self-identified and formally diagnosed autistic individuals in this data set. We followed the Strengthening the Reporting of Observational Studies in Epidemiology Statement guidelines in writing this article.^20^

## Literature Overview

Previous research into TNB autistic health care has tended to use a medical framework that pathologizes both experiences of TNB identity and autism.^21,22^ Pyne^23^ elaborated on this, in his exploration of the historical connections between TNB and autistic research, and the ways in which Applied Behavioral Analysis for autism effectively aligns with the practice of conversion therapy for trans and queer children. It has been observed that both political or ideological anti-trans campaigners, and some researchers, appear to be motivated to maintain a medical model of health care that defines both autism and gender variance in terms of deficit and overcautiously metes out access to GAC.^24,25^ Autistic individuals, for instance, sometimes experience greater barriers to GAC due to extended (or even perpetual) waiting periods,^26,27^ and misattribution or minimization of TNB identity to a mere characteristic of autism.^28,29^

Autistic TNB individuals—perhaps partially due to ableist and transphobic discrimination—also appear to disproportionately experience poorer access to primary and mental health care, and more negative health outcomes.^30^ This is consistent with findings that, compared with their cisgender and heterosexual peers, lesbian, gay, bisexual, queer and TNB autistic people individually experience greater health disparities and difficulty finding competent and respectful primary and mental health care providers.^31–33^ The existence of autistic TNB individuals has, in this context, been rhetorically deployed by those who argue that GAC should be rigidly gatekept and made unavailable to autistic people (see Rodriguez^34^ and Strang and Fischbach^35^ for further discussion of this).

Autistic TNB self-advocates and the neurodiversity movement have pushed back against these barriers to care, by arguing that autism and the experience of being TNB are natural elements of human diversity, rather than disorders.^36^ Furthermore, existing research theorizes that the observed overlap between autism and TNB identity may occur because autistic individuals are simply less likely to accede to cultural expectations that they conform to gendered norms.^22,37^ As in the field of autism research,^38^ some autistic TNB self-advocates and communities have called for a shift away from research on the etiology of TNB identities and autism co-incidence, and toward a focus on community-based research that addresses the needs and priorities of autistic TNB people themselves.^39,40^

## Methods

### Sample and procedures

Trans PULSE Canada data on TNB individuals were collected throughout the summer and fall of 2019, through a community-based research study of TNB health and lived experience. A convenience sample was recruited online through social media and mailing lists, in-person at Pride festivals and other community events, and through peer research associates available in 11 major Canadian cities. Participants self-selected to take either the full (∼70 minutes) or short-form (∼10 minutes) survey.^15^ This survey and all questions within it were pilot tested “for clarity and for functionality (p. E1215).”^15^ There were no restrictions on participation, beyond the inclusion criteria and survey completion deadline.

Eligibility criteria included living in Canada; being 14 years or older; identifying their gender as differing in some way from their sex assigned at birth; and providing consent to participate. Surveys were available in English and French and were completed online, on paper, by telephone, or with the assistance of a peer research associate. The full Trans PULSE Canada survey and further details on study methodology are available in existing publications.^15,19^ Research ethics boards at Western University, Unity Health Toronto, and Wilfrid Laurier University reviewed and approved this study.

The total sample included 2873 participants, of whom 2481 completed the full-length survey and 392 completed the short-form survey. The present article compares the participants who reported a diagnosis of autism or self-identify as autistic with those who are allistic.

### Measures

#### Independent variable

Our key independent variable, “autism status,” was organized into three categories: those diagnosed with autism, self-identified autistic individuals, and allistic individuals. Two questions from the survey were used to determine autism status: “Have you been diagnosed with any of the following?” and “Do you self-identify as someone who currently lives with the following realities or conditions?” All who checked the option “autism or Asperger's” in the first question were included in the “diagnosed with autism” category. All those who checked the option “autistic” in the second question (but did not indicate an autism diagnosis in the first) were included in the “self-identified autistic” category. All other participants were included in the allistic category. Note that the question on autism self-identity was asked of all participants, including those who indicated that they were diagnosed with autism. As a result, we have included 18 individuals who indicated that they were diagnosed with autism but did not identify as autistic.

#### Covariates

The following self-reported demographic variables were also measured: age, sexual orientation, educational attainment, personal annual income, employment situation, household income, number of people supported on that income, sex assigned at birth, current gender identity, co-occurring disability identities and diagnoses, a place of birth outside of Canada (being an immigrant), Indigenous identity, identifying as a person of color, being perceived or treated as a person of color, and living in a rural area. Sexual orientation was measured by asking participants to self-select from a list of sexualities that also included a space to write in a label not already defined. Racialized persons were coded as those who either identified as (or were perceived or treated as) people of color. Low-income household was coded for participants aged 25 years and older, and according to household income and number of people supported, using Statistics Canada's low-income measure threshold.^41^

#### Dependent variables

While Trans PULSE Canada represents an independent study, several methodological variables were drawn from Statistics Canada's Canadian Community Health Survey to allow for comparison to the general population. These include self-rated overall health and mental health, having a primary health care provider, and unmet health care needs.

Self-rated overall health and mental health are widely used measures of health status that have good reliability and validity.^42–45^ They were assessed by asking “in general would you say your health is…?” and “in general, would you say your mental health is…?” Options for both questions were “excellent,” “very good,” “good,” “fair,” and “poor.” We mitigated against the risk that autistic respondents may have interpreted the subjective terms “fair” and “poor” differently from allistic respondents, by including autistic individuals in the disability priority population consultation team (PPCT) that helped to determine survey questions.

Dependent variables not drawn from Statistics Canada include those related to GAC (such as current GAC status, barriers delaying this care, and having had a mental health assessment to access it in the past year). If participants indicated experiencing the latter, we then asked whether the assessment was helpful or harmful, and if they had avoided sharing information about their mental health or autism to access the desired care.

Additional dependent variables came from check-all-that-apply questions that were developed by the Trans PULSE research team to inquire into potential TNB-specific positive and negative experiences with primary and mental health care providers. There were 7 positive and 12 negative items listed for experiences with primary care providers, and 6 positive and 11 negative items for experiences with mental health care providers. Positive and negative items were intermixed in the checklists and not marked as such on the survey. These included positive items such as the provider using inclusive forms, using the participant's correct name and pronouns, and demonstrating knowledge of TNB-related health concerns.

Negative items included the provider repeatedly misgendering the participant, telling them the practitioner did not know enough about TNB-related care to provide it, and using hurtful or insulting language about this group. We summed the responses (Yes = 1, No = 0) separately over positive and negative items for, respectively, experiences with primary and mental health care providers. This resulted in determining the sum of positive experiences with primary care providers, the sum of negative experiences with primary care providers, the sum of positive experiences with mental health care providers, and the sum of negative experiences with mental health care providers.

Note, however, that the number of participants who completed the questions about experiences with primary and mental health care is much smaller than the total analytic sample. Furthermore, these questions were only asked of those who had a primary care provider and/or had accessed mental health care in the past year.

### Statistical analyses

All analyses were conducted using SAS 9.4. (SAS Institute, Inc.). Frequencies of demographic and health status variables were estimated for each autism status group. As demographic variables and key health outcomes were asked of all participants, regardless of whether they completed the short- or full-length survey, these analyses were unweighted. We similarly estimated frequencies for TNB-specific positive and negative experiences with primary and mental health care providers across autism status groups. Because these questions were not included in the short version of the survey, proportions (%) were weighted to represent the demographic distribution of all participants.^15^ To assess for potential differences in demographics and primary and mental health care experiences across the three comparison groups (diagnosed with autism, undiagnosed but self-identified autistic, and allistic), we conducted the Pearson chi-square tests on unweighted data and Rao–Scott chi-square tests on weighted data.

Finally, logistic regression analyses were conducted for key health outcome variables. The three comparison groups were dummy-coded, with the allistic group being the reference. Odds ratios and adjusted odds ratios (AORs) were estimated from univariate and multivariable logistic regression models, respectively. For example, if the AOR for reporting having fair/poor general health is 2.44 among diagnosed autistics, it indicates that they have 2.44 times the odds of reporting fair/poor (as opposed to excellent/very good) general health, compared with allistics, after adjusting for the covariates included in the multivariable model. The multivariable models were based on a directed acyclic graph and were adjusted for age, Indigenous identity, racialization, immigration status, rural residence, and sex/gender categories. The latter were coded as sex assigned at birth (male or female) and self-reported primary gender identity (man or boy, woman or girl, Indigenous or other cultural gender identity, and nonbinary, genderqueer, agender, or a similar identity).

For the sums of positive and negative health care experiences (see dependent variables), we tested the hypothesis that the autistic groups (diagnosed or self-identified) tended to have fewer positive experiences, and more negative experiences, with primary and mental health care providers. We categorized each sum of health care experiences (the dependent variable) into three categories: sum = 0, 1, and ≥2, and conducted multinomial logistic regression analyses. We used sum ≥2 as the reference category for positive experiences and sum = 0 as the reference category for negative experiences.

## Results

Of all participants in the Trans PULSE Canada study (*n* = 2873), 15 were excluded from our analysis due to missing data on autistic identity and diagnosis. Of the included 2858 participants, 230 (8.1%) reported that they had been diagnosed with autism, and 176 (6.2%) reported self-identifying as autistic. These two autistic categories were mutually exclusive. Together, these 406 autistic individuals represent 14.3% of all included participants. The remaining 2452 participants (85.7%) were allistic.

### Demographic findings

We found key demographic differences between autistic and allistic participants on most demographic variables (Table 1). Compared with allistic participants, autistic individuals averaged slightly younger, were more likely to identify as asexual or bisexual, and less likely to identify as straight. This group was also more likely to report a physical, developmental, or learning disability other than autism. Overall, autistic participants reported lower levels of educational attainment, full-time employment, personal annual incomes, and were more likely to be living in a low-income household than were allistic respondents. Self-identified and diagnosed autistic participants were broadly alike demographically, although self-identified participants were more likely to be older than 19 years and to identify as “non-binary, genderqueer, agender, or a similar gender identity”* than those with a diagnosis.

**Table 1.** Participant Demographics by Autism Status

	Diagnosed with autism (*n* = 230)		Self-identified autistic (*n* = 176)		Allistic (*n* = 2452)		Total	*p*
	n	%	n	%	n	%		
Age^a^ (years)								
14–19	50	21.7	13	7.5	303	12.4	366	**<0.001**
20–24	56	24.4	60	34.5	503	20.6	619	
25–34	87	37.8	72	41.4	891	36.4	1050	
35–49	30	13.0	23	13.2	512	20.9	565	
50+	7	3.0	6	3.5	237	9.7	250	
Missing	0		2		6		8	
Sexual orientation^b^								
Asexual	57	24.8	59	33.5	267	10.9	383	**<0.001**
Bisexual	67	29.1	72	40.9	670	27.3	809	**<0.001**
Gay	27	11.7	31	17.6	305	12.4	363	0.12
Lesbian	43	18.7	31	17.6	367	15.0	441	0.23
Pansexual	67	29.1	57	32.4	764	31.2	888	0.76
Queer	108	47.0	107	60.8	1258	51.3	1473	**0.02**
Straight	5	2.2	4	2.3	204	8.3	213	**<0.001**
Two-spirit	13	5.7	10	5.7	92	3.8	115	0.19
Unsure/questioning	20	8.7	11	6.3	216	8.8	247	0.51
Other	14	6.1	13	7.4	84	3.4	111	**0.006**
Missing	0		1		9		10	
Sex assigned at birth^a^								
Male	79	36.2	49	28.7	773	33.2	901	0.29
Female	139	63.8	122	71.4	1557	66.8	1818	
Missing	12		5		122		139	
Current gender identity^a^								
Man or boy	56	25.6	25	14.5	609	26.0	690	**0.01**
Woman or girl	52	23.7	38	22.0	571	24.4	661	
Indigenous or other cultural gender identity (e.g., Two-Spirit)	6	2.7	6	3.5	46	2.0	58	
Nonbinary, genderqueer, agender, or a similar identity	105	48.0	104	60.1	1116	47.7	1325	
Missing	11		3		110		124	
Disability^b^,^c^								
Mental health condition	203	88.3	161	91.5	1611	65.7	1975	**<0.001**
Learning disability	85	37.0	50	28.4	327	13.3	462	**<0.001**
Chronic pain	57	24.8	35	19.9	338	13.8	430	**<0.001**
Chronic illness	53	23.0	39	22.2	301	12.3	393	**<0.001**
Blind or visually impaired	36	15.7	30	17.1	205	8.4	271	**<0.001**
Mobility or physical disability	41	17.8	20	11.4	148	6.0	209	**<0.001**
Intellectual/developmental disability	39	17.0	14	8.0	59	2.4	112	**<0.001**
Acquired brain injury	12	5.2	9	5.1	65	2.7	85	**0.02**
Deaf or hearing loss	11	4.8	3	1.7	43	1.8	57	**0.007**
Missing	0		0		0		0	
Education^d^								
High school diploma or less	20	16.1	19	18.8	181	11.1	220	**<0.001**
Some college or university or CEGEP	41	33.1	30	29.7	332	20.3	403	
College or university degree, or CEGEP degree	49	39.5	39	38.6	802	49.1	890	
Graduate or professional degree	14	11.3	13	12.9	320	19.6	347	
Missing	0		0		5		5	
Personal annual income^a^,^d^								
<$15,000	52	43.0	41	41.4	370	23.0	463	**<0.001**
$15,000–$29,000	35	28.9	24	24.2	384	23.8	443	
$30,000–$49,000	18	14.9	21	21.2	365	22.6	404	
$50,000–$79,000	9	7.4	10	10.1	296	18.4	315	
$80,000+	7	5.8	3	3.0	197	12.2	207	
Missing	3		2		28		33	
Employment^a^^d^								
Permanent full-time	29	27.9	29	32.6	624	45.1	682	**<0.001**
Employed not permanent full-time	37	35.6	29	32.6	478	34.5	544	
Not employed or on leave	30	28.9	29	32.6	192	13.9	251	
Not employed and student or retired	8	7.7	2	2.3	91	6.6	101	
Missing	20		12		255		287	
Low-income household^a^^d^	72	61.0	53	57.6	621	38.9	746	**<0.001**
Missing	6		9		42		57	
Indigenous^a^	32	13.9	20	11.4	200	8.2	252	**0.006**
Missing	0		0		5		5	
Immigrant^a^	26	11.4	17	9.7	325	13.3	368	0.31
Missing	2		1		8		11	
Racialized^a^	31	13.5	30	17.1	341	13.9	402	0.50
Missing	0		0		3		3	
Lives in rural area^a^	14	6.5	7	4.1	156	6.6	177	0.44
Missing	13		5		74		92	

All percentages are calculated excluding the missing participants for each variable. Boldface indicates statistical significance.

^a^ For variables with mutually exclusive options, we conducted an overall chi-square test and reported only one *p*-value.

^b^ Participants could choose more than one option, so the percentages sum to >100%. Participants were marked as missing if they did not select any of the options, including the “other” category. For variables in which participants could choose more than one option, we conducted a chi-square test for each option and thus reported multiple *p*-values.

^c^ Participants were marked as missing if they did not select any of the options. However, participants must have answered this question to be included in the analytic sample, as the autism variable was derived from this question. As such, no participants in the analytic sample were missing.

^d^ Of participants 25 years of age and older.

### General health

Autistic participants reported statistically significant worse overall general health and well-being than their allistic peers (Table 2). Nearly half of both self-identified (47.0%) and diagnosed (43.9%) autistic, compared with one quarter (24.1%) of allistic, participants reported fair or poor general health. Self-identified autistic participants had 4.40 times the adjusted odds (95% confidence interval [CI]: 2.75–6.98) of reporting fair or poor general health (rather than excellent or very good health), compared with allistic participants, while those with an autism diagnosis had 2.44 times the adjusted odds (95% CI: 1.69–3.52).

**Table 2.** Key Health Outcomes by Autism Status as Predictor

Individual key health outcomes	Predictor						
	Diagnosed with autism, n = 230			Self-identified autistic, n = 176			Allistic (Ref.), n = 2452
	%	OR (95% CI)	AOR (95% CI)	%	OR (95% CI)	AOR (95% CI)	%
General Health Self-Rating^a^							
Excellent/very good (Ref.)	27.3	1.00	1.00	16.9	1.00	1.00	39.1
Good	28.8	1.12 (0.77–1.62)	1.00 (0.67–1.48)	36.1	**2.28 (1.44**–**3.60)**	**2.20 (1.33**–**3.49)**	36.8
Fair/poor	43.9	**2.61 (1.85**–**3.68)**	**2.44 (1.69**–**3.52)**	47.0	**4.53 (2.90**–**7.07)**	**4.40 (2.75**–**6.98)**	24.1
Does not have primary health care provider^b^	19.6	1.06 (0.74–1.52)	0.95 (0.65–1.4)	24.1	1.38 (0.95–2.00)	1.19 (0.80–1.77)	18.7
Unmet health care need in past 12 months^c^	54.8	**1.63 (1.23**–**2.17)**	**1.50 (1.11**–**2.04)**	60.9	**2.09 (1.51**–**2.90)**	**1.79 (1.27**–**2.52)**	42.6
Mental Health Self-Rating^d^							
Excellent/very good (Ref.)	10.0	1.00	1.00	3.2	1.00	1.00	17.8
Good	22.0	1.34 (0.78–2.31)	1.22 (0.68–2.19)	24.4	**4.63 (1.81**–**11.86)**	**3.79 (1.46**–**9.85)**	29.1
Fair/poor	68.0	**2.27 (1.40**–**3.69)**	**1.79 (1.05**–**3.05)**	72.4	**7.55 (3.06**–**18.64)**	**5.73 (2.28**–**14.39)**	53.1
Gender-Affirming Medical Care Status^e^							
Had all desired treatment (Ref.)	18.0	1.00	1.00	17.7	1.00	1.00	26.9
In the process of completing treatment	35.0	**1.69 (1.12**–**2.55)**	**1.55** (1.00–2.40)	38.6	**1.89 (1.20**–**2.99)**	**1.67 (1.03**–**2.70)**	31.1
Planning to receive treatment, but have not begun	21.8	**2.42 (1.53**–**3.82)**	**1.83 (1.09**–**3.08)**	18.4	**2.06 (1.20**–**3.52)**	**1.96 (1.11**–**3.48)**	13.6
Not sure if going to seek treatment	11.7	1.51 (0.88–2.57)	1.28 (0.68–2.39)	12.7	1.66 (0.92–3.00)	1.00 (0.52–1.91)	11.6
Not planning to receive treatment	13.6	1.21 (0.73–2.01)	1.10 (0.61–1.97)	12.7	1.14 (0.64–2.06)	0.78 (0.41–1.47)	16.8

Results represent a series of multinomial or binomial logistic regression analyses with various health outcomes and autism status being the sole predictor. Outcomes are listed in the rows and the predictor in the columns to reduce table width. Allistic is the reference group. Regarding AOR (adjusted odds ratio), we adjusted for age, Indigenous identity, racialization, immigration status, rural residence, and sex/gender categories coded from sex assigned at birth and gender identity. % unweighted percent; OR: odds ratio; CI: confidence interval. Boldface indicated statistical significance.

^a^ Missing: 18 participants diagnosed with autism, 10 self-identified autistic participants, and 216 allistic participants.

^b^ Missing: 21 participants diagnosed with autism, 14 self-identified autistic participants, and 241 allistic participants.

^c^ Homecare was excluded, as only asked of those aged 50 years and older. Missing: 22 participants diagnosed with autism, 15 self-identified autistic participants, and 250 allistic participants.

^d^ Missing: 30 participants diagnosed with autism, 20 self-identified autistic participants, and 339 allistic participants.

^e^ Missing: 24 participants diagnosed with autism, 18 self-identified autistic participants, and 306 allistic participants.

We failed to find a statistically significant difference between the likelihood that autistic and allistic participants had a primary care provider. However, we did find that autistic participants reported significantly higher rates of unmet health care needs. Overall, 54.8% of participants with an autism diagnosis, 60.9% of self-identified autistic participants, and 42.6% of allistic participants reported an unmet health care need other than home care services^†^ in the past year. Self-identified autistic participants had 1.79 times (95% CI: 1.27–2.52) and diagnosed individuals had 1.50 times (95% CI: 1.11–2.04) the adjusted odds of reporting an unmet health care need compared with allistic participants.

### Mental health

We found statistically significant mental health disparities between autistic and allistic participants (Table 2). This was especially true among those who self-identified as autistic. In all, 68.0% of diagnosed, 72.4% of self-identified, and 53.1% of allistic participants reported “fair” or “poor” mental health.

Self-identified participants had 5.73 times (95% CI: 2.28–14.39) and diagnosed participants had 1.79 times (95% CI: 1.05–3.05) the adjusted odds of reporting fair or poor (rather than excellent or very good) mental health, compared with allistic participants. However, as the CI for self-identified autistic participants is quite wide and overlaps with the CI for participants with an autism diagnosis, the true difference in fair or poor mental health between these groups may not be as large as these findings suggest.

### Gender-affirming care

Only 3.7% of diagnosed and 1.1% of self-identified autistic participants reported being denied GAC due to being autistic (Table 3). However, among those who had a mental health or readiness assessment for GAC in the past year, 25.5% of those diagnosed with autism and 36.1% of those self-identified as autistic indicated that they avoided sharing information about autism during the assessment to access this care.

**Table 3.** Health Care Experiences by Autism Status

	Diagnosed with autism (*n* = 118)		Self-identified autistic (*n* = 108)		Allistic (*n* = 1185)		Total	*p*
	n	%	n	%	n	%		
In the past 12 months, a primary health care provider has …^a^–^c^								
Used correct name, pronouns, or gendered language	83	60.9	80	73.3	920	65.6	1083	0.13
Open to discussing TNB-related health concerns	81	58.9	77	70.3	887	63.2	1045	0.19
Asked about name or pronouns	48	34.9	44	40.5	451	32.3	543	0.19
Demonstrated knowledge of TNB-related health concerns	46	33.7	44	39.8	583	41.5	673	0.21
Needed you to educate them about TNB-related health concerns	45	32.2	43	39.7	398	28.1	486	**0.03**
Used TNB-inclusive forms	43	31.4	40	36.3	470	33.6	553	0.72
Advocated for you as a TNB person	35	26.0	32	29	387	27.4	454	0.87
Took steps to make physical exams more comfortable for you as a TNB person	32	23.9	35	31.9	405	28.8	472	0.36
Told you they don't know enough about TNB-related care to provide it	33	23.8	25	22.3	220	15.5	278	**0.01**
Repeatedly misgendered	27	19.5	24	22.6	234	16.7	285	0.22
Thought name or gender on ID/forms was a mistake	13	9.3	9	8	74	5.4	96	0.11
Refused to discuss TNB-related health concerns	9	6.4	7	6.2	34	2.5	50	**0.006**
Told you that you were not really TNB	5	3.9	1	0.9	15	1	21	**0.02**
Discouraged exploring gender	4	2.8	3	2.7	21	1.5	28	0.37
Used hurtful or insulting language about TNB people	3	2.5	4	3.5	46	3.3	53	0.88
Insisted on examining irrelevant body parts	3	2.1	4	3.5	20	1.5	27	0.28
Belittled or ridiculed you as a TNB person	2	1.8	0	0	16	1.2	18	n/a^f^
Refused to see you or ended care because you were TNB	2	1.8	2	1.8	19	1.4	23	0.88
Refused to examine parts of body because you are TNB	1	0.7	1	0.9	16	1.2	18	0.86
Missing	0		0		2		2	
In the past 12 months, a mental health care provider has …^a^,^c^,^d^								
Used correct name, pronouns, or gendered language	82	69.4	81	74.9	879	74.1	1042	0.52
Open to discussing TNB-related health concerns	71	60.3	68	62.4	755	63.6	894	0.77
Asked about name or pronouns	63	53.1	62	58.0	616	51.9	741	0.47
Used TNB-inclusive forms	57	48.6	42	38.6	522	43.9	621	0.33
Demonstrated knowledge of TNB-related health concerns	50	42.9	40	37.0	549	46.4	639	0.15
Advocated for you as a TNB person	38	32.6	30	27.7	366	30.6	434	0.73
Needed you to educate them about TNB-related health concerns	31	25.9	42	40.0	319	26.8	392	**0.01**
Repeatedly misgendered you	23	19.2	22	20.8	159	13.6	204	**0.047**
Wasn't able to separate mental health concerns from TNB identity	17	14.2	20	19.3	131	11.1	168	**0.03**
Told you they don't know enough about TNB-related care to provide it	11	9.2	17	16.6	119	9.9	147	**0.09**
Used hurtful or insulting language about TNB people	7	5.9	9	8.4	53	4.5	69	0.18
Discouraged exploring gender	7	5.8	5	4.9	42	3.6	54	0.40
Refused to discuss TNB-related health concerns	5	4.3	6	5.7	27	2.2	38	0.05
Told you that you were not really TNB	5	4.1	4	3.6	33	2.8	42	0.66
Thought name or gender on ID/forms was a mistake	4	3.4	9	8.5	50	4.4	63	0.14
Belittled or ridiculed you for being TNB	2	1.7	2	1.7	21	1.8	25	>0.99
Refused to see you or ended care because you were TNB	1	0.9	2	1.7	13	1.1	16	0.79
Missing	0		0		1		1	
Gender-affirming medical care experiences^a^,^c^,^e^								
Barriers delaying gender-affirming medical care								
Can't afford treatment	48	45.6	27	33.0	266	32.5	341	**0.03**
Can't afford travel to treatment	28	26.2	13	16.5	176	21.4	217	0.29
Denied because of gender identity or expression	4	3.8	2	2.8	21	2.5	27	0.77
Denied because of weight	9	8.4	7	7.8	50	5.9	66	0.51
Denied because of mental health	8	7.5	3	3.3	41	4.9	52	0.38
Denied because of autism	4	3.7	1	1.1	0	0.0	5	n/a^f^
Denied because of disability	6	5.5	1	1.1	9	1.2	16	**0.003**
On a waitlist	36	34.1	29	33.2	323	38.6	388	0.45
Other	22	20.3	25	29.2	195	23.2	242	0.33
Missing	11		4		129		144	
Rating of experience of mental health assessment^g^,^h^								
Helpful	10	36.3	6	25.5	135	49.0	151	0.34
Harmful	3	10.8	3	12.9	21	7.6	27	
Both helpful and harmful	7	28.1	5	23.1	54	19.1	66	
Neither helpful nor harmful	7	24.8	9	38.5	69	24.4	85	
Missing	0		0		0		0	
Avoiding sharing information about … to access desired care^a^,^c^,^g^								
Mental health	10	38.4	10	42.8	89	32.1	109	0.51
Autism	6	25.5	8	36.1	1	0.4	15	**<0.001**
Other	0	0.0	3	14.9	9	3.2	12	n/a^f^
Missing	1		0		2		3	

*p*-Values from the Rao–Scott chi-square tests. Unweighted *n* weighted %. Boldface indicates statistical significance. n/a, not applicable; TNB, transgender and nonbinary.

^a^ Participants could choose more than one option, so the percentages sum to >100%.

^b^ Of participants who currently had a primary health care provider. This includes 147 participants diagnosed with autism, 114 self-identified autistic participants, and 1532 allistic participants.

^c^ For variables in which participants could choose more than one option, we conducted a chi-square test for each option and thus reported multiple *p*-values.

^d^ Of participants who had accessed mental health care in the past 12 months. This includes 118 participants diagnosed with autism, 108 self-identified autistic participants, and 1185 allistic participants.

^e^ Of participants who were planning on, or in the process of completing, gender-affirming medical care. This includes 117 participants diagnosed with autism, 90 self-identified autistic participants, and 958 allistic participants.

^f^ *p*-Value could not be calculated due to zero observations in one of the categories.

^g^ Of participants who had a mental health assessment for gender-affirming medical care in the past 12 months. This includes 27 participants diagnosed with autism, 23 self-identified autistic participants, and 279 allistic participants.

^h^ For this mutually exclusive variable, we conducted an overall chi-square test and reported only one *p*-value.

Participants with an autism diagnosis (45.6%) were more likely than both self-identified (33.0%) and allistic (32.5%) participants to have difficulty affording GAC. Similarly, diagnosed participants (5.5%) were more likely to be denied GAC due to a disability other than autism than self-identified autistic (1.1%) or allistic (1.2%) individuals.

Autistic participants were also more likely to be in the process of completing or planning (but had not yet begun) GAC than those who were allistic (Table 2). That is, when asked if they were “planning to receive treatment, but have not begun,” they were less likely than allistic participants to have completed all desired GAC. Specifically, self-identified autistic participants had 1.67 times the adjusted odds (95% CI: 1.03–2.70) of being in the process of completing GAC (e.g., starting hormones, pursuing surgery) and 1.96 times the odds (95% CI: 1.11–3.48) of planning but having not begun it compared with allistic participants. Similarly, those with an autism diagnosis had 1.83 times the odds (95% CI: 1.09–3.08) of planning but having not begun to receive GAC than allistic participants.

### Experiences of primary and mental health care

As shown in Table 3, autistic participants were more likely to report that a mental health care provider had repeatedly misgendered them (20.8% of self-identified and 19.2% of diagnosed autistic participants vs. 13.6% of allistic participants). Autistic respondents were also more likely to report that a mental health care provider was unable to separate their mental health and TNB identity (19.3% of self-identified participants and 14.2% of diagnosed participants vs. 11.1% of allistic participants). Finally, self-identified autistics were more likely to report needing to educate both their primary (39.7% of self-identified participants and 32.2% of diagnosed participants vs. 28.1% of allistic participants) and mental (40.0% of self-identified participants and 25.9% of diagnosed participants vs. 26.8% of allistic participants) health care providers about their TNB-related needs. However, given the exploratory nature of this research, we did not adjust for multiple comparisons. As such, it is possible that these findings are false positives.

As shown in Table 4, self-identified autistic participants were more likely than allistic participants to report two or more (as opposed to zero) negative experiences with primary and mental health care providers (primary care: AOR = 1.88, 95% CI: 1.16–3.06; mental health care: AOR = 1.87, 95% CI: 1.14–3.06). However, this group was also less likely to report zero (as opposed to two or more) positive experiences with primary care providers (AOR = 0.53; 95% CI: 0.30–0.96). Overall, we failed to find statistically significant differences between allistic and diagnosed autistic participants regarding the number of positive and negative experiences with primary and mental health care providers.

**Table 4.** Transgender and Nonbinary-Specific Health Care Experiences in Past 12 Months, Weighted Frequencies and Odds Ratios by Autism Status as Predictor

	Predictor						
	Diagnosed with autism (*n* = 118)			Self-identified autistic (*n* = 108)			Allistic (*n* = 1185)
	%	OR (95% CI)	AOR (95% CI)	%	OR (95% CI)	AOR (95% CI)	%
TNB-specific experiences with primary health care provider in past 12 months^a^							
Number of positive experiences^b^							
None	25.6	1.26 (0.83–1.91)	1.12 (0.69–1.82)	17.3	0.71 (0.42–1.21)	**0.53 (0.30**–**0.96)**	22.7
One	14.0	1.58 (0.92–2.68)	1.41 (0.80–2.49)	10.8	1.02 (0.53–1.95)	0.89 (0.46–1.75)	9.9
Two or more (Ref.)	60.4	1.0		71.9	1.00		67.4
Number of negative experiences^c^							
None (Ref.)	48.7	1.00		44.5	1.00		55.6
One	26.9	1.20 (0.78–1.83)	1.14 (0.73–1.77)	24.8	1.21 (0.74–1.99)	1.13 (0.67–1.89)	25.6
Two or more	24.4	1.48 (0.95–2.31)	1.31 (0.82–2.10)	30.8	**2.05 (1.28**–**3.27)**	**1.88 (1.16**–**3.06)**	18.8
TNB-specific experiences with mental health care provider in past 12 months^d^							
Number of positive experiences^b^,^c^							
None	17.6	1.04 (0.62–1.73)	1.12 (0.65–1.91)	13.8	0.77 (0.43–1.38)	0.66 (0.35–1.24)	17.7
One	12.1	1.41 (0.77–2.59)	1.32 (0.68–2.54)	11.8	1.30 (0.69–2.44)	1.28 (0.66–2.49)	8.97
Two or more (Ref.)	70.2	1.00		74.4		1.00	73.3
Number of negative experiences^b^,^c^							
None (Ref.)	60.1	1.00		45.5		1.00	62.0
One	20.7	1.13 (0.69–1.84)	1.18 (0.71–1.94)	26.3	**1.89 (1.15**–**3.09)**	1.57 (0.93–2.67)	18.9
Two or more	19.2	1.03 (0.62–1.71)	0.91 (0.53–1.57)	28.2	**2.01 (1.24**–**3.25)**	**1.87 (1.14**–**3.06)**	19.1

Results represent a series of multinomial logistic regression analyses with autism status being the sole predictor. Allistic is the reference group. Outcomes are listed in the rows and the predictor in the columns to reduce table width. Regarding AOR (adjusted odds ratio), we adjusted for age, Indigenous identity, racialization, immigration status, rural residence, and sex/gender categories coded from sex assigned at birth and gender identity. %; unweighted percent; OR: odds ratio; CI: confidence interval. Boldface indicated statistical significance.

^a^ Of participants who currently had a primary health care provider. This includes 147 participants diagnosed with autism, 114 self-identified autistic participants, and 1532 allistic participants. Two allistic participants had missing data for these questions.

^b^ For the number of positive experiences, having two or more positive experiences is the reference category.

^c^ For the number of negative experiences, having none is the reference category.

^d^ Of participants who had accessed mental health care in the past 12 months. This includes 118 participants diagnosed with autism, 108 self-identified autistic participants, and 1185 allistic participants. One allistic participant was missing data for these questions.

## Discussion

This article reports on our research into autistic TNB individual's experiences of primary, mental health, and gender-affirming health care, using data collected in the 2019 Trans PULSE Canada Study.^19^ Autistic respondents to the Trans PULSE Canada Study had lower overall incomes, educational attainment, and were less likely to be employed than allistic participants. Socioeconomic status is known to substantially impact health, including the autistic TNB population,^5–7,10^ and increased support in these areas (particularly if it is created by and for autistic adults) may help to mitigate poor health care outcomes in the areas of gender and TNB-related health care.^40^

Autistic respondents in this study also had poorer experiences of primary and mental health care, compared with both allistic respondents and the general Canadian population (as represented by recent Statistics Canada data), and experienced greater barriers accessing GAC than allistic respondents.^46–48^ They were, for instance, more likely to be in the process of completing or planning GAC than allistic respondents, who were more likely to have completed all desired GAC. Very few participants reported being denied GAC due to being autistic. However, many indicated that they preemptively hid their autism from providers to avoid the possibility of being denied this care.

The lower degree to which autistic respondents in our study had completed all desired GAC may be because they were, on average, younger than allistic respondents. However, this may also result from their providers suspecting autism despite their proactively hiding it, and/or interpreting autistic traits (like difficulty responding to indirect questions) as uncertainty about the desire for GAC.^49,50^ In this case, denial of care due to autism would be indirect and not necessarily explicitly communicated or obvious and might result in longer or even indeterminate waiting periods rather than outright refusal.^49^ This is supported by clinical and research narratives that question the authenticity of autistic TNB identity,^33^ ascribe it to rigid and fixed thinking,^51^ dispute autistic individuals' inherent capacity to consent to GAC,^52^ and advise increased scrutiny and longer waiting periods before allowing it for this population.^26,29,53,54^ As observed in our study, autistic TNB people sometimes report that providers conflate the relationship between their TNB identity and autism.^21,49,54,55^

It has also been observed that some autistic individuals may be more likely to openly identify as TNB, due to a tendency not to perceive or care about the social conditioning that might prevent them from becoming aware of or expressing this identity.^37,54^ Our finding that self-identified autistic individuals may be more likely to identify as nonbinary is in line with a greater openness to gender identity exploration.^55–57^ Ultimately, clinicians should take care to listen to and understand everyone's unique experience of their gender identity and autism.^28,32,33^

While our findings were not always statistically significant, self-identified autistic participants appeared to experience worse overall health, greater unmet health care needs, and more negative experiences with primary and mental health care providers than those with a diagnosis. These participants were more likely to report that primary and mental health care providers were unable to distinguish between their mental health and TNB identity and that they needed to educate providers on TNB-related concerns. While there are little data comparing the adverse health care experiences of self-identified and diagnosed autistic individuals, that which exists has failed to find statistically significant differences between them.^12,16^ Most mentions of self-identified autistic individuals in the literature appear to relate to either exploring the phenomenon of autistic self-identification itself^17,18^ or indicate excluding or cleaning these participants from data sets.^58^

Although the Trans PULSE data set did not focus directly on clinical practice recommendations, our subanalysis did reveal areas in which it could be improved. For instance, autistic TNB individuals frequently encounter misgendering from primary and mental health care practitioners while seeking GAC and tend to hide their autism from these practitioners. Guidance in making one's practice more autism and TNB friendly can be found in the studies by Gratton^59^ and Kourti,^60^ which detail how to support autistic TNB people from the perspective of individuals at this intersection. Both highlight the importance of improving trauma-informed practice, understanding differential experiences of gender, dealing with minority stress, and improving the accessibility of GAC. Similarly, autistic rights organizations have created guides to help autistic people access and navigate this type of health care.^61^

Finally, it is likely that many of the 6.2% of Trans PULSE participants who self-identified as autistic had difficulty accessing autism support services (such as in education and aspects of daily living) that typically require a diagnosis. In any case, a recent report by the Canadian Academy of Health Sciences found that government-funded supports for autistic adults are “essentially non-existent,” difficult to access, and often open only to autistic people with co-occurring intellectual disabilities (p. 193).^7^ They recommend improving overall health outcomes by basing service access on need, rather than diagnosis. However, while basing service access on need alone would certainly benefit autistic TNB individuals, provider transphobia might continue to undermine their access to and experience of autism services. Furthermore, the financial and pragmatic barriers to obtaining an autism diagnosis, particularly as an adult, are high.^62^ The assessment process, for instance, can cost several thousand dollars, is not always covered by health insurance, and can be very difficult to access through the public health care system.^7,63,64^

### Study strengths

One strength of this data set is that it consists of a large, community-based sample that introduces some of the first data about the experiences of autistic TNB people in Canada. Another is that we took the relatively unusual step of including self-identified autistic individuals in our analysis. Self-identification is common within the autistic community,^65^ due in part to the expense and difficulty of obtaining an autism diagnosis, particularly in adulthood.^66^ Garfield and Yudell have argued that the exclusion of self-identified autistic individuals from research may “violate the principle of participatory justice as it could undermine the inclusivity of a community partnership and lead to greater exclusion of those with the least access to competent diagnostic services (p. 456).”^67^ The substantial barriers to health care, negative health outcomes, and socioeconomic marginalization experienced by self-identified autistic participants in our sample highlight the unmet needs of this group, and the importance of including them in future research and policy initiatives.

### Study limitations

From a quantitative perspective, the primary limitation of our study is that our sample was not random and thus cannot be assumed to represent all TNB individuals or populations. For instance, while the full survey reported that 14% of our 2873 participants were autistic, this does not mean that 14% of all TNB people in Canada are. Furthermore, our study only examined negative primary and mental health care outcomes in relation to being TNB. Participants may, however, have experienced positive or negative interactions with providers related to autism, accessibility needs, or other social identities and memberships not captured in our data. For example, communication challenges, executive function, and/or sensory challenges all contribute to an autistic person's experience of worse overall health, compared with their allistic peers.^68^

Finally, we did not measure the number of participants' visits to mental health or primary care providers. However, it is possible that some individuals accessed these types of health care more often than others and, as a result, had greater opportunities for both positive and negative experiences. Indeed, we noted the apparently unusual situation where self-identified autistic participants were more likely than allistic participants to report both negative and positive experiences with primary health care providers. This may account for these observed differences. Similarly, we did not ask autistic participants whether they felt that they were more or less likely to have been asked to attend a mental health assessment in the past year.

### Future research

As noted, it can be quite difficult to obtain an adult diagnosis of autism and, as a result, self-identified autistics may experience compounded barriers to health care access not experienced by those with a diagnosis. More research into this possibility and on this intersection is clearly needed. On the other hand, it is possible that individuals with a diagnosis are more likely to experience health care conservatorship^69,70^ and so be hindered in making decisions regarding their access to GAC. Future research should thus also explore the potential impact of conservatorship on enabling or hindering access to GAC. Other avenues for research include exploring autistic TNB individuals' resilience in the TNB community, of primary and mental health care, and in gaining access to GAC.

## Conclusions

Using questions created by the Trans PULSE Canada's disability PPCT, we were able to compare both TNB respondents who are autistic with those who are allistic, as well as those who self-identify as autistic versus those who have a diagnosis. Our analysis revealed some similarities and several key differences between these groups. Autistic participants, for instance, were more likely to report a non-autistic disability, had worse overall health, greater unmet health needs, and more negative experiences accessing care than allistic participants.^31–33^ Self-identified autistic participants further reported a higher rate of needing to educate primary and mental health care providers, and being misgendered by mental health care provides, compared with both allistic and diagnosed autistic groups. Finally, while very few autistic respondents reported being denied GAC explicitly due to autism (3.7% of diagnosed and 1.1% of self-identified autistics), 25.5% of those diagnosed with and 36.1% of self-identified autistic respondents proactively avoided disclosing autism with GAC practitioners to access this care, due to concern they might face such a barrier.

These findings and observations highlight the need for changes regarding the treatment of TNB autistics. Where we have observed greater health challenges and discrimination in this population, there is an opportunity to course correct and change practice to better account for their experiences. We have observed a population that is highly capable of identifying their needs and pursuing goals regarding GAC. We hope that future research will explore the resilience of autistic TNB individuals, as well as further investigate the unique experiences of those who self-identify as autistic, especially considering the high economic and organizational costs associated with obtaining a diagnosis. Regardless, all future research should include autistic TNB people throughout the process, from conception through dissemination.^71^

## Notes

^*^
This was the specific wording in the Trans PULSE Canada survey.^19^

^†^
All Trans PULSE PPCT were empowered to create questions that would only apply to respondents from their group. The questions on home care services were developed by the older adult PPCT and, as a result, an older autistic adult might be asked about this question, but not all autistic individuals would have been.
